# An unexpected cause of sudden cardiac arrest in a young swimmer

**DOI:** 10.1002/joa3.12530

**Published:** 2021-03-27

**Authors:** Xabier Cia Mendioroz, Eusebio Garcia‐Izquierdo, Susana Mingo Santos, Victor Castro‐Urda, Jorge Toquero‐Ramos, Ignacio Fernandez‐Lozano

**Affiliations:** ^1^ Cardiology Unit Hospital Universitario Puerta de Hierro Majadahonda Madrid Spain

**Keywords:** coronary aneurysms, Kawasaki disease, sudden cardiac arrest

## Abstract

Sudden cardiac arrest (SCA) is an uncommon but devastating event among young adults. While inherited cardiomyopathies and channelopathies represent an important proportion of sudden deaths, coronary artery disease remains a significant contributor in this age group. ECG findings are essential to guide the first steps of diagnostic work‐up of SCA, but sometimes can overlap between different etiologies. In this article we present a 16‐year‐old female who experienced SCA during vigorous swimming whose ECG was compatible with long QT syndrome. However, evaluation of the coronary anatomy provided the diagnosis of Kawasaki disease.

## Graphical abstract

Sudden cardiac arrest is uncommon among young adults. Inherited cardiomyopathies and channelopathies are the most frequently identified cardiac etiologies. However, assessment of coronary artery anatomy is mandatory in order to rule out coronary artery anomalies.

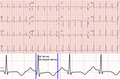

## 1 ECG FINDINGS ARE ESSENTIAL TO GUIDE THE FIRST STEPS OF THE DIAGNOSTIC WORK‐UP OF SCA

We present the initial ECG after SCA of a 16‐year‐old female, with no relevant past medical or family history, who was admitted to our intensive cardiac care unit (ICCU) after experiencing SCA during vigorous swim practice. Cardiopulmonary resuscitation was promptly initiated and return of spontaneous circulation was achieved after a single shock using an automated external defibrillator (AED). ECG tracings were later retrieved from the AED, showing the presence of ventricular fibrillation (VF) as the initial shockable rhythm. Cardiovascular and neurological status was normal upon arrival.

**FIGURE 1:**
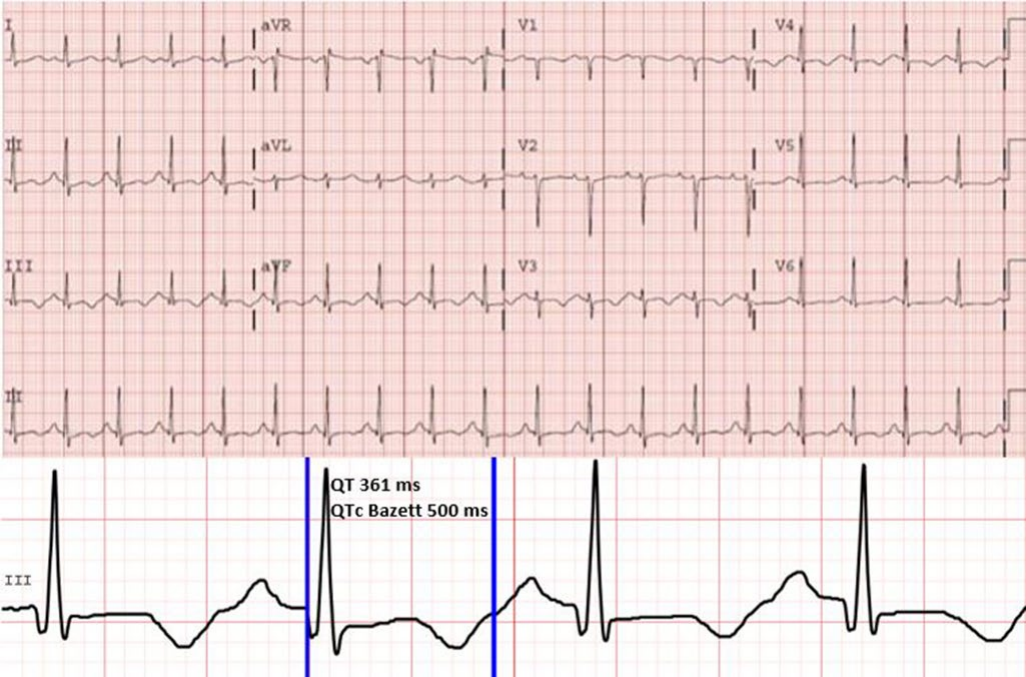
Initial ECG at the ICCU showing QTc up to 500 ms and slight ST depression and negative T waves in V3, V4, and the inferior leads

A marked QT prolongation could be seen on initial ECGs, with a QTc up to 500 ms in the absence of electrolyte imbalance or QT prolonging drugs. Additionally, minor changes in the ST segment and T‐wave inversion in the inferior leads were also observed (Figure 1). Initial blood tests were normal, except for slightly elevated troponin I levels. Transthoracic echocardiography performed at the ICCU showed mild reduction of left ventricular ejection fraction without segmental wall‐motion abnormalities. This was thought to be due to myocardial stunning after defibrillation and left ventricular ejection fraction was normalized on echocardiography performed on subsequent days.

**FIGURE 2:**
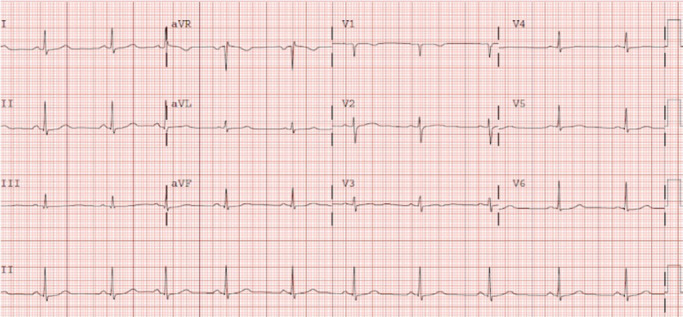
ECG after a few days of admission. QTc interval normalized without further repolarization changes

The patient remained stable and the ECG normalized after a few days (Figure 2).

**FIGURE 3:**
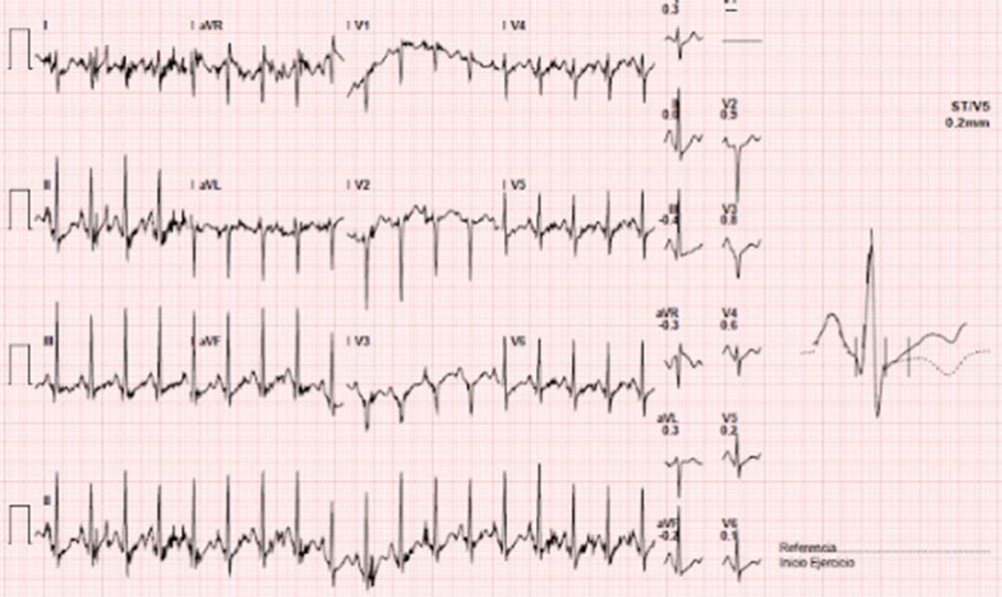
Submaximal treadmill stress test achieving 90% of maximum predicted heart rate. Image corresponds to peak exertion, without observing ST segment or T‐wave alterations respect to baseline ECG

Further evaluation was performed to look for additional causes of cardiac arrest, including flecainide test that was normal. During treadmill stress test, we found a paradoxical lengthening of the QTc with exercise and at recovery phase without any ST segment changes (Figure 3). Interestingly, genetic testing for long‐QT syndrome (LQTS) was negative in our patient. Besides QT measurement, transthoracic echocardiography was performed during stress test. Wall motion abnormalities at peak exercise were observed, suggesting the presence of ischemia in the territory of left anterior descending artery.

**FIGURE 4:**
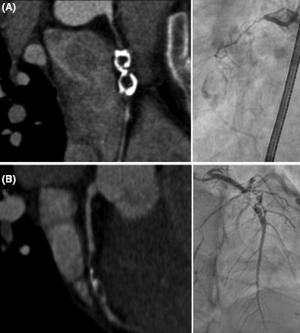
Coronary computed tomography angiography and coronary angiography showing a giant calcified aneurysm of the right coronary artery (RCA) (A) and a severe stenosis in the mid segment of left anterior descending artery (LAD) (B)

Finally, coronary computed tomography angiography (CCTA) was performed in order to assess the anatomy of the coronary vessels. CCTA revealed the presence of giant calcified aneurysms causing a chronic total occlusion in the proximal portion of the right coronary artery and a severe stenosis in the midsegment of the left anterior artery. These findings were corroborated by invasive coronary angiography (Figure 4) and were compatible with the diagnosis of Kawasaki disease (KD) with coronary artery sequelae. At that point, her parents reported that she had suffered an episode of high fever, severe rash and skin desquamation at the age of 3 that required hospital admission.

The case was discussed by the heart team in our hospital. Since myocardial ischemia related to KD sequelae was considered to be the most likely cause of SCA, the patient finally underwent coronary artery bypass grafting (CABG) and received a subcutaneous implantable cardioverter defibrillator (S‐ICD) for secondary prevention. She recovered uneventfully and was discharged home 20 days after surgery maintaining the treatment with nadolol that was initiated during admission.

Kawasaki disease is a vasculitis of unknown etiology that generally occurs during the infancy and childhood. It is typically a self‐limited condition, with fever and other acute inflammatory manifestations (ie, rash, conjunctivitis, mucositis). One of the major cardiovascular complications of KD is the development of coronary artery aneurysms (CAAs), which can go unnoticed until adult life if they were not diagnosed during the initial episode. KD represents the most common cause of CAAs in childhood and the second most common cause in adults, right after atherosclerosis. However, some other less frequent conditions should be included in the differential diagnosis, such as Takayasu arteritis, systemic lupus erythematosus, fibromuscular dysplasia, connective tissue disorders, or infectious diseases.

Until now, there are no clear guidelines for the diagnosis and treatment of adult patients. Thus, KD patients diagnosed with coronary aneurysm or myocardial infarction in adult life should be treated and followed in the same way as patients with such conditions associated with etiologies other than KD and the management of cardiovascular risk factors is highlighted.

The evidence of QT prolongation in the first ECGs along with the fact that cardiac arrest occurred while swimming initially raised the suspicion of LQTS. Interestingly, prolongation of the QT interval has been also described as an early sign of transmural ischemia, which may also explain the paradoxical lengthening of the QTc during exercise that was seen in our patient. However, despite the negative result of the genetic test, we feel that LQTS could not be completely ruled out. Regardless of the true etiology of SCA in this case, the decision to start beta‐blocker treatment and implant an S‐ICD was made in order to reduce the likelihood of potentially lethal cardiac events in the future. The treadmill stress test was not repeated after surgery, which could have provided a valuable assessment of the result of CABG and a better understanding of the true etiology of SCA

## 2 CONCLUSION

In the present case, coronary imaging was key to complete the evaluation of SCA in a young patient without previously known structural heart disease. This article highlights the possible influence of ischemia on the QT interval and coronary imaging to improve the diagnostic approach in young adults after SCA.

## CONFLICT OF INTEREST

None.

